# Spinal Calcrl^+^ neurons amplify mechanical itch signaling via synaptic plasticity in chronic itch model

**DOI:** 10.1371/journal.pone.0336113

**Published:** 2025-11-17

**Authors:** Huifeng Jiao, Qi Dai, Zhaoting Li, Min Yuan, Guoqun Xu, Yingxin Tian, Guangyuan Su, Yu Zhang, Chong Sun, Shunqi Wang, Chaolin Ma, Haili Pan

**Affiliations:** 1 School of Basic Medical Sciences, Jiangxi Medical College, Nanchang University, Nanchang, Jiangxi, China; 2 Institute of Pain Medicine and Special Environmental Medicine, Co-Innovation Center of Neuro regeneration, Nantong University, Nantong, Jiangsu, China; 3 The Second Affiliated Hospital, Institute of Biomedical Innovation, Jiangxi Province Key Laboratory of Brain Science and Brian Health, and School of Basic Medical Sciences, Jiangxi Medical College, Nanchang University, Nanchang, Jiangxi, China; 4 Neurological Institute of Jiangxi Province and Department of Neurology, Jiangxi Provincial People’s Hospital, The First Affiliated Hospital of Nanchang Medical College, Nanchang, Jiangxi, China; University of Nebraska Medical Center College of Medicine, UNITED STATES OF AMERICA

## Abstract

Chronic itch, a devastating dermatological disorder, lacks targeted therapies due to incomplete understanding of its neural circuitry. Building on seminal studies that identified neuropeptide Y (NPY) inhibitory interneurons and their downstream urocortin 3-positive (Ucn3^+^)/Y1R-expressing neurons, calcitonin receptor-like receptor-positive (Calcrl^+^) neurons, identified as spinal projection neurons, have been proposed to contribute to mechanical itch signaling, though their underlying mechanistic role remains undefined. In the present study, using chemogenetic manipulation, behavioral tests, morphological assays and electrophysiological approaches in allergic contact dermatitis, atopic dermatitis and Psoriasis chronic itch models, we elucidates the role of spinal Calcrl^+^ neurons in mechanical itch pathophysiology. We report that: (1) Chemogenetic activation of spinal Calcrl^+^ neurons induces enhanced mechanical itch sensitization and increased spontaneous scratching behaviors in naïve mice; (2) Chemogenetic inhibition of spinal Calcrl^+^ neurons alleviates mechanical itch sensitization and spontaneous scratching behaviors in chronic itch models; (3) Chronic itch enhances intrinsic excitability of Calcrl+ neurons in chronic itch model; (4) Aβ-fiber-evoked synaptic excitation of Calcrl+ neurons is significantly amplified in chronic itch, accompanied by reduced inhibitory input. Our study elucidates a pathological synaptic plasticity mechanism in chronic itch, wherein spinal Calcrl+ neurons undergo hyperexcitability, enhanced Aβ-fiber-evoked excitatory transmission and reduced inhibitory input. These findings establish a spinal Calcrl-dependent circuit as a critical driver of mechanical itch sensitization, providing actionable targets for disrupting maladaptive itch circuits in dermatological disorders.

## Introduction

Itch, an unpleasant somatosensory sensation that drives scratching behavior, serves as a critical evolutionary defense mechanism against environmental threats through acute responses. However, chronic itch remains one of the most debilitating dermatological conditions worldwide, characterized by persistent spontaneous pruritus and continuous scratching that exacerbates skin damage, significantly impairing quality of life [1,2]. Despite its clinical prevalence, chronic itch pathophysiology remains poorly understood, with current treatments showing limited efficacy due to the multifactorial nature of its underlying mechanisms [3–5].

Mechanical itch, induced by hair follicle vibration, represents a distinct pruritic subtype that exhibits remarkable plasticity in chronic conditions [6]. Recent studies have demonstrated that mechanical itch sensitivity is enhanced in chronic itch patients, suggesting aberrant signal processing in peripheral and central nervous systems [4,7].

Neuropeptide Y (NPY), a 36-residue neuromodulator conserved across vertebrates, regulates fundamental processes including energy homeostasis, emotional behavior, and cardiovascular function via Gi-coupled receptors (Y1, Y2, Y4, Y5) [8–10]. Activation of these receptors suppresses cAMP signaling, modulating synaptic transmission and neural plasticity [9,11]. Within the spinal cord, NPY and its receptors (notably Y1/Y2) exhibit concentrated expression in superficial dorsal horn laminae (I-II) – regions critical for sensory integration. This strategic localization underpins NPY’s significant role in modulating sensory processing, including pain and itch [10,12,13]. Substantial preclinical work, notably a series of studies by Bradley K. Taylor and colleagues, has demonstrated that spinal NPY alleviates neuropathic pain through specific activation of the NPY1R receptor [12,14,15].

Pioneering studies by Bourane and Duan et al. (2015) established a fundamental framework for NPY’s role in mechanical itch signaling. Through genetic manipulation of NPY::Cre mice, they demonstrated that spinal NPY^+^ neurons form a dedicated “gate control” pathway for mechanical itch [16]. Building on this foundation, our group and Prof. Martyn Goulding’s team simultaneously made seminal advances in 2019: While we identified spinal urocortin 3 (Ucn3)^+^ excitatory neurons as critical downstream effectors of this inhibitory gate [17], Acton et al. (2019) clarified that neuropeptide Y1 receptor-expressing neurons constitute the primary effector population in this pathway, where NPY-Y1 signaling suppresses mechanical itch stimuli [18]. However, critical questions remain unresolved regarding how mechanical itch signals transmitted to higher brain regions.

The spinal Calcitonin receptor-like receptor-positive (Calcrl^+^) neurons, a subset of excitatory interneurons localized in the superficial spinal dorsal horn (SDH; laminae I-II), are critical intermediaries in pain processing, particularly within pathological contexts such as inflammation and chronic pain [19]. By forming a functional complex with receptor activity-modifying protein 1 (RAMP1), these neurons act as high-affinity receptors for calcitonin gene-related peptide (CGRP), amplifying excitatory synaptic transmission and enhancing central sensitization—key mechanisms underlying the development and maintenance of chronic pain hypersensitivity [20–23]. Ren et al. (2024) identified spinal Calcrl^+^ neurons as essential relay neurons in the transmission of mechanical itch signals to parabrachial nucleus (PBN) [24]. However, their functional contribution to chronic itch remains poorly understood.

Our current study further investigates this pathway, suggesting that functional manipulation of Calcrl^+^ neurons plays a pivotal role in mechanical itch sensitization. Specifically, activation of these neurons induces scratching behaviors in naïve mice, while inhibition alleviates chronic itch symptoms in various models.

This research offers three-dimensional innovation and synthesizes seminal findings from the pioneering study [16], our 2019 dual-pathway discovery [17,18], and the recent advances by Ren et al. (2024) [24]. Moreover, it provides critical mechanistic insights into how chronic itch disrupts ascending pruritic pathways. Given that current therapies fail to target the underlying neural circuitry, this study holds transformative potential for developing mechanism-based therapeutics aimed at specific nodes within the pruritic circuit.

## Materials and methods

### Animals

In this study, 8–10-week-old, 20-25g male wild-type C57BL/6J mice were purchased from Henan Sikebes Biotechnology Co., Ltd. All mice were housed under SPF (Specific Pathogen Free) conditions. Mice were housed in cages with a 12-hour light/dark cycle, with free access to food and water. The room temperature was maintained at 22 ± 0.5°C, with no more than 5 mice per cage.

### Ethics statement

All experimental protocols involving animals were approved by Institutional Animal Care and Use Committee of Nanchang University (Approval No. NCULAE-20221228064) and Institutional Animal Care and Use Committee of Nantong University (Approval No. S20210311-013), and the care and use of all mice was conducted in strict accordance with the National Institutes of Health Guide for the Care and Use of Laboratory Animals (8th ed.) and AVMA Guidelines (2020). Postoperative analgesia was exclusively administered for survival surgeries: subcutaneous meloxicam (5 mg/kg) delivered immediately after surgery and repeated at 24-hour intervals for 48 hours. Non-survival procedures required no postoperative analgesia as these were terminal experiments performed under deep anesthesia.

All suffering minimization protocols were implemented as follows: Isoflurane inhalation served as the anesthetic agent (5% induction, 2–2.5% maintenance), with continuous perioperative monitoring of pedal withdrawal reflex and respiratory rate. Mandatory euthanasia criteria included weight loss exceeding 20%, mobility impairment preventing basic functions, or unrelieved distress persisting beyond 24 hours. The institutional veterinarian reviewed all animals twice weekly.

For euthanasia, behavioral cohorts underwent gradual-fill CO₂ asphyxiation at 30% chamber volume displacement per minute followed by immediate cervical dislocation after respiratory arrest, with death verification via absent corneal and pedal reflexes. Terminal procedures utilized sustained isoflurane anesthesia with bilateral thoracotomy confirmation prior to perfusion.

All personnel involved in animal procedures, including anesthesia administration, surgery, behavioral testing, and endpoint assessment, received comprehensive training in animal handling, species-specific techniques, aseptic surgery practices, anesthesia monitoring, euthanasia protocols, and recognition of signs of pain and distress. This training was provided and certified by the institutional veterinarian and the Institutional Animal Care and Use Committee (IACUC) prior to initiating any experimental work. No animals required early euthanasia.

### Chronic itch models establishment

The Allergic Contact Dermatitis (ACD) model, Atopic Dermatitis (AD) Model and Psoriasis (PSO) model were established according to previously published protocols [17].

The ACD model was induced by 3,4-dimethoxy-3-cyclobutene-1,2-dione (Dinitrofluorobenzene, DNFB). Briefly, mice underwent abdominal and dorsal hair removal (about 4 cm² area) 3 days prior to baseline behavioral assessments. Following a 3-day acclimatization period, baseline scratching behavior was recorded. For sensitization, 35 μl of 1% DNFB dissolved in acetone was topically applied to the shaved skin for three consecutive days. After a 5-day rest phase, the challenge phase commenced with daily application of 35 μl DNFB solution to the inner ear. Mechanical itch was quantified using 0.07 g von-Frey filament after three days of application and spontaneous scratching behavior was monitored after six consecutive days’ challenge.

The AD Model was induced by calcipotriol. Briefly, five days before treatment, the fur on the nape of the neck was shaved, and baseline measurements of mechanical itch scores and spontaneous scratching bouts were recorded two days prior to model induction. Mice were treated topically with 2 nmol of calcipotriol once daily for eight days on the left ear skin, using 20 μL of ethanol as a vehicle.

The PSO Model was induced via topical application of 5% imiquimod (IMQ). Following hair removal from the nuchal region (about 2 cm² area) five days prior to treatment initiation, baseline mechanical itch scores and spontaneous scratching responses were recorded 48 hours before model induction. Under standardized conditions, mice received daily topical applications of 5% IMQ emulsion (20 μL volume), using vaseline as vehicle, administered consecutively over eight days.

### Spontaneous and mechanical itch behavior recording

Mice were individually placed in transparent compartments and allowed to acclimate for at least 15 minutes before video recording for 30 minutes. Scratching behavior was quantified by counting the number of times the mouse lifted its hind paw to the model site and lowered it to the mouth or floor. Mechanical itch sensitivity was assessed using von Frey filaments (0.07g). The hind paw response to mechanical stimuli was considered a positive response. The percentage of positive responses to five separate stimuli was calculated to indicate mechanical itch sensitization. Behavioral assessments were performed using a double-blind experimental design to minimize observer bias, with experimenters blinded to group assignments during testing.

Behavioral tests lasted for 26–32 days, immediately following completion of behavioral testing and associated data collection, mice were transported to a dedicated procedure room. All mice were humanely euthanized within one-hour post-behavioral assessment.

### Stereotaxic viral injection into the C2-C3 spinal cord

C57BL/6J mice were anesthetized with isoflurane (5% induction via chamber, 2–2.5% maintenance via nose cone) and positioned in a stereotactic frame (RWD Life Science Co., 68044). Anesthetic depth was continuously verified through pedal reflex suppression and respiratory pattern stability. A syringe pump was then used to inject 300nL of virus at 4 sites in the dorsal horn of the C2-C3 or L4-L5 spinal cord at a rate of 30nL/min using a 10 µL microsyringe. rAAV-Calcrl-CRE-WPREs combined with AAV5-hSyn-DIO-tdTomato-WPREs, AAV5-hSyn-DIO-hM4Di-tdTomato-WPREs, or rAAV-hSyn-DIO-hM3Dq-tdTomato-WPREs (Wuhan Sarmt) were used to express tdTomato, hM4Di or hM3Dq in spinal Calcrl^+^ neurons. Viruses were diluted to ~5 × 10¹¹ GC/mL in PBS for sparse labeling.

Following viral injection, postoperative analgesia was administered via subcutaneous meloxicam (5 mg/kg) with monitoring continuing for 72 hours. Daily weight measurements and wound assessments were performed, with heating pads maintaining 37°C ambient temperature until full ambulation. Animals received soft-diet supplementation for 24 hours and underwent twice-daily wound inspection; euthanasia was mandated if autotomy or >10% weight loss occurred.

### Spinal cord slice preparation

For acute spinal cord slice preparation for electrophysiology, mice aged P23-30 were deeply anesthetized using inhaled isoflurane (5% for induction in oxygen, maintained at 2–2.5% via nose cone) to ensure a complete absence of nociceptive reflexes (confirmed by lack of response to firm toe pinch). Immediately following confirmation of deep anesthesia, animals were rapidly decapitated using sharp surgical scissors. Then the cervical or lumber spinal cord attached with dorsal roots was quickly removed and placed in ice-cold, modified cutting solution containing the following components (in mM): 80 NaCl, 2.5 KCl, 1.25 NaH_2_PO4, 0.5 CaCl_2_, 3.5 MgCl_2_, 25 NaHCO_3_, 75 sucrose, 1.3 sodium ascorbate, and 3.0 sodium pyruvate. The pH was adjusted to 7.2, and the osmolality was maintained between 310–320 mOsm. This solution was continuously oxygenated with a mixture of 95% O_2_ and 5% CO_2_. Using a vibratome (Leica VT1000S), parasagittal spinal cord slices of 480 μm thickness were cut. Following this, the slices were incubated for about 1 hour at 33°C in artificial cerebrospinal fluid (ACSF) containing (in mM): 125 NaCl, 2.5 KCl, 2 CaCl_2_, 1 MgCl_2_, 1.25 NaH_2_PO4, 26 NaHCO_3_, 10 D-glucose, 1.3 sodium ascorbate, and 3.0 sodium pyruvate. The ACSF had a pH of 7.4, osmolality of 310–320 mOsm, and was bubbled with 95% O_2_ and 5% CO_2_.

Whole-Cell Patch Clamp Recordings: Whole-cell patch-clamp recordings were performed at 32°C on submerged spinal cord slices in ACSF, which was continuously perfused at 3 ml/min. After the incubation period, the slices were transferred to a recording chamber for subsequent recordings from Calcrl-tdTomato-expressing dorsal horn neurons, Aβ-evoked responses were recorded as previously described [17]. Borosilicate glass pipettes (Sutter Instruments, Novato, CA) with a resistance of 3–6 MΩ were used. The pipettes were filled with an internal solution containing the following (in mM): 130 potassium gluconate, 5 KCl, 4 Mg_2_ATP, 0.5 NaGTP, 10 HEPES, and 0.5 EGTA. The solution pH was adjusted to 7.28 using KOH, and its osmolality was maintained at 310–320 mOsm. Data were collected with a MultiClamp 200B patch-clamp amplifier and Digidata 1440B (Molecular Devices, San Jose, CA). The responses were low-pass filtered at 2 kHz and digitized at 5 kHz. Offline analysis was performed using pClamp 10.4 software (Molecular Devices, San Jose, CA).

### Immunofluorescence assay

As a non-survival terminal procedure, transcardial perfusion was conducted under sustained isoflurane anesthesia with pedal reflex abolition confirmation. Death during perfusion was verified by bilateral thoracotomy before tissue processing. Briefly, following deep anesthessia with inhaled isoflurane, mice were immediately subjected to transcardial perfusion. Perfusion consisted of 0.1M phosphate-buffered saline (PBS, pH 7.4) followed by 4% paraformaldehyde (PFA), with death occurring during the exsanguination phase under sustained anesthesia. No animals regained consciousness. The C2-C3 spinal cord segments were then dissected, post-fixed in the 4% PFA for 5–6 hours at 4°C, and thoroughly rinsed in ice-cold 0.1M PBS to remove residual fixative. Tissue was placed in 20% sucrose solution overnight and then in 30% sucrose until it sank to the bottom of the test tube. Transverse sections (18µm) of the spinal cord were cut using a cryostat (LEICA CM1950), collected, and stored at −20°C. Sections were incubated with primary antibodies overnight at 4°C (Calcrl, c-Fos), washed, and then incubated with secondary antibodies (Alexa 568 and Alexa 488 conjugated secondary antibodies, 1:500, Invitrogen) at room temperature for 1.5 hours. After washing, the sections were counterstained with DAPI (1:10,000, Invitrogen) and mounted with anti-fluorescence fading mounting medium (S2100, Solarbio). Images were captured using an Olympus VS200 digital slide scanner and an Olympus FV1000 laser confocal microscope. Sholl analysis and cell counting were performed using ImageJ software.

### Statistical analysis

The data presented in this study are expressed as mean ± SEM. Statistical analysis was performed using Prism (GraphPad 9.0.0). The significance of differences between experimental groups was evaluated using the two-tailed unpaired Student’s t-test, chi-square test or repeated-measures two-way ANOVA followed by Sidak’s post hoc analyses, with p < 0.05 considered statistically significant.

## Results

### Spinal Calcrl+ neurons mediate mechanical itch signal transmission

**Fig 1:**
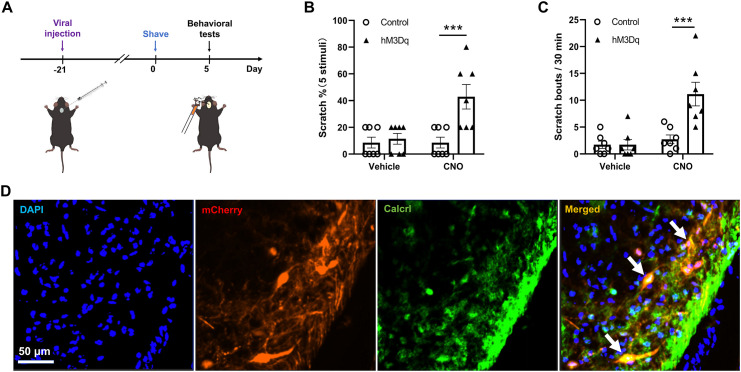
Chemogenetic modulation of spinal Calcrl^+^ neurons alters mechanical itch responses. **(A)** Experimental strategy: rAAV-Calcrl-CRE-WPREs was co-injected with AAV5-hSyn-DIO-hM4Dq-tdTomato (hM4Dq) or AAV5-hSyn-DIO-tdTomato (Control) into the spinal cord of C57 mice to selectively activate Calcrl^+^ neurons via chemogenetics. Behavioral tests were performed three weeks later. **(B)** CNO-induced activation of Calcrl^+^ neurons significantly increased mechanical itch responses (n = 7 mice/group). Baseline responses were comparable between groups, while hM4Dq mice exhibited significant increase in sensitivity post-CNO. **(C)** Spontaneous scratching episodes were elevated in hM4Dq mice following CNO treatment (n = 7 mice/group). **(D)** Representative immunofluorescence image showing colocalization of Calcrl (green) and AAV5-hSyn-DIO-hM4Dq-tdTomato (red) in spinal neurons (arrows). Behavioral data are displayed as mean ± SEM. *** indicated p < 0.001 as determined by repeated-measures two-way ANOVA, followed by Sidak’s post hoc tests. Tissue sections from 3 mice (9 sections total) were processed for immunofluorescence staining.

To investigate the functional role of spinal Calcrl^+^ neurons in mechanical itch, we selectively manipulated their activity using chemogenetics. Intraspinal injections of rAAV-Calcrl-CRE-WPREs combined with AAV5-hSyn-DIO-hM4Dq-tdTomato or control viruses (AAV5-hSyn-DIO-tdTomato) were performed in C57 mice (experimental schematic shown in Fig 1A). Behavioral testing revealed no significant differences in baseline mechanical itch responses between groups under vehicle conditions (Control: 8.57 ± 4.04%, hM4Dq: 10.00 ± 4.14%; p = 0.9268). However, activation of Calcrl^+^ neurons via clozapine-N-oxide (CNO) significantly augmented mechanical itch sensitivity in hM4Dq mice compared to controls (Control: 8.57 ± 4.04%, hM4Dq: 40.00 ± 9.56%; p = 0.0006; Fig 1B).

Spontaneous scratching behavior mirrored these effects: baseline scratching bouts showed no difference between the two groups (Control: 1.71 ± 0.68 bouts/30 min, hM4Dq: 1.71 ± 0.97 bouts/30 min; p > 0.9999), whereas CNO treatment markedly elevated scratching frequency in hM4Dq mice (Control: 2.71 ± 0.81 bouts/30 min, hM4Dq: 11.14 ± 2.20 bouts/30 min; p = 0.0003; Fig 1C). These data indicate that spinal Calcrl^+^ neuron activation amplifies both evoked mechanical itch responses and spontaneous scratching behaviors.

Immunofluorescent validation confirmed colocalization of Calcrl immunoreactivity with AAV5-hSyn-DIO-hM4Dq-tdTomato in spinal neurons (Fig 1D), verifying virus-specific targeting of Calcrl^+^ neurons.

### Spinal Calcrl+ neurons contribute to chronic itch pathogenesis

**Fig 2:**
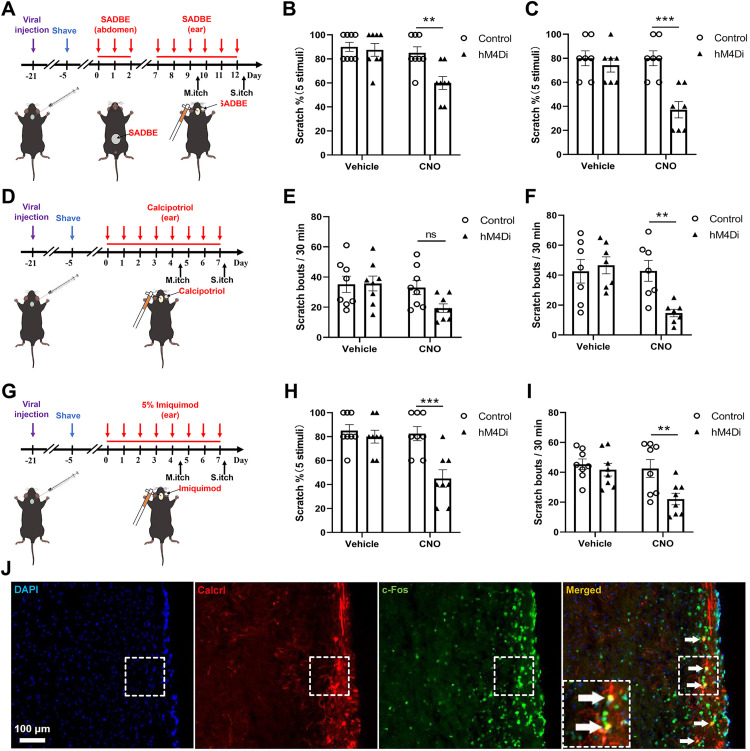
Chemogenetic inhibition of Calcrl^+^ neurons attenuates chronic itch in multiple chronic itch models. **(A-C)** Allergic Contact Dermatitis (ACD) Model (n = 7 mice for each group): Experimental timeline showing intraspinal viral injection, ACD model induction, and behavioral testing phases **(A)**; Quantification of mechanical itch responses revealed significant CNO-mediated suppression in hM4Di mice, with no effect in vehicle-treated controls **(B)**; Spontaneous scratching frequency decreased following CNO administration **(C)**. **(D-F)** Atopic Dermatitis (AD) Model (n = 8 mice for each group): Schematic illustrating viral delivery, AD model induction protocol, and behavioral assessment schedule **(D)**; Mechanical itch sensitization was reduced in hM4Di mice versus controls **(E)**; Scratching activity decreased post-CNO **(F)**. (G-I) Psoriasis (PSO) Model (n = 8 mice for each group): Experimental workflow demonstrating viral injection, PSO model induction, and behavioral evaluation **(G)**; Mechanical itch sensitivity decreased following CNO treatment **(H)**; Spontaneous scratching frequency reduced by CNO treatment **(I)**; c-Fos Validation. **(J)** Representative immunofluorescence showing colocalization (white arrows) of Calcrl^+^ neurons (red) with c-Fos activation (green) in ACD mice post-CNO. Data represent mean ± SEM. The statistical significance of intergroup differences was assessed using repeated-measures two-way ANOVA, followed by Sidak’s post hoc analyses for behavioral experiments. Data are presented as mean ± SEM (ns: not significant, **p < 0.01, ***p < 0.001). Immunofluorescence quantification was performed on 9 sections derived from 3 mice.

To assess the role of spinal Calcrl^+^ neurons in chronic itch, we established three distinct chronic itch models in C57 mice expressing AAV5-hSyn-DIO-hM4Di: allergic contact dermatitis (ACD), atopic dermatitis (AD) and Psoriasis (PSO). Chemogenetic inhibition of Calcrl+ neurons via CNO significantly attenuated mechanical itch hypersensitivity and spontaneous scratches in all models (Fig 2).

In ACD mice, CNO treatment reduced mechanical responses from 80.00 ± 6.17% (Control) to 37.14 ± 6.80% (hM4Di, p = 0.001), while vehicle-treated controls showed no change: Control group: 80.00 ± 6.17%, hM4Di group: 74.29 ± 5.71%, p = 0.7721 (Fig 2B). Similarly, spontaneous scratching bouts in ACD mice decreased from 42.86 ± 6.96 bouts/30 min (Control) to 14.71 ± 2.50 bouts/30 min (hM4Di, p = 0.0065 0.01) after CNO (Fig 2C), with vehicle-treated controls showed no change (42.57 ± 7.85 bouts/30 min vs. 46.57 ± 5.66 bouts/30 min, p = 0.8751).

AD mice exhibited a comparable reduction in mechanical itch sensitivity, with values decreasing from 85.00 ± 5.00% (Control:) to 60.00 ± 5.34% (hM4Di; p = 0.0023; Fig 2E). Additionally, spontaneous scratching bouts decreased from 33.13 ± 4.76 (Control) to 19.38 ± 2.86 (hM4Di), showing a notable downward trend despite lacking statistical significance (p = 0.0852; Fig 2F).

Notably, no significant differences were observed under baseline conditions (vehicle-treated groups) (spontaneous scratching: Control: 35.25 ± 5.41 vs. hM4Di: 35.75 ± 4.95 scratches/30 min, p = 0.9963; mechanical itch: Control: 90.00 ± 3.78 vs. hM4Di: 87.50 ± 5.26 scratches/30 min, p = 0.9218; Figs 2E and 2F), confirming the specificity of Calcrl-mediated modulation.

In PSO mice, mechanical responses decreased from 82.50 ± 5.90% (Control) to 45.00 ± 7.32% (p = 0.0002) post-CNO, with no change in vehicle treatment: Control group: 85.00 ± 5.00, Di group: 80.00 ± 5.35, p = 0.8043 (Fig 2H);

Spontaneous itch in PSO mice, with vehicle treatment, Control: 45.38 ± 3.50, Di: 41.75 ± 4.22, p = 0.8172; with CNO treatment, Control: 42.50 ± 5.99, Di: 21.13 ± 3.83, p = 0.0066 (Fig 2I).

In the ACD model, we examined the activation status of Calcrl^+^ neurons using immunofluorescent histochemical staining. Our results revealed a significant colocalization of spinal Calcrl^+^ neurons with c-Fos expression (Fig 2J).

### Enhanced intrinsic excitability of spinal Calcrl+ neurons in chronic itch models

**Fig 3:**
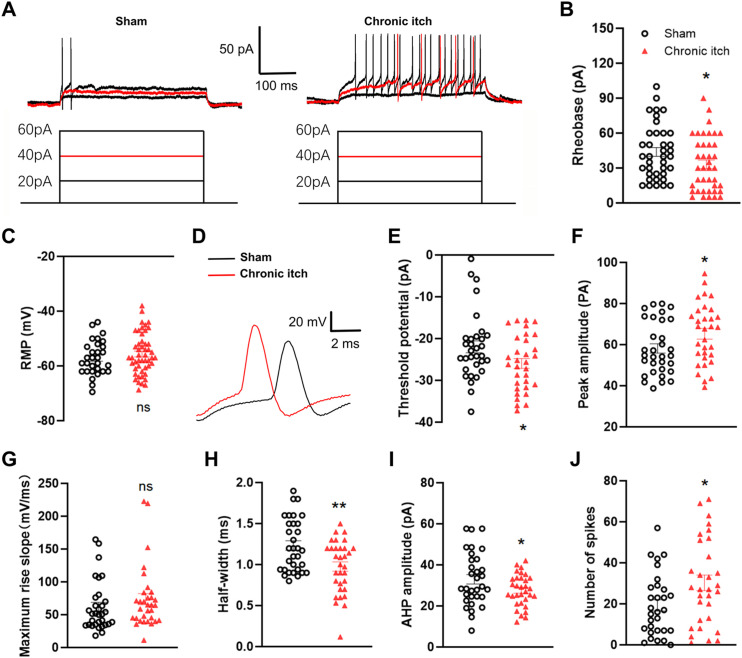
Enhanced intrinsic excitability of spinal Calcrl^+^ neurons in chronic itch. **(A)** Representative step depolarization responses from Calcrl^+^ neurons in both group. **(B)** Rheobase measurements revealed hyperexcitability in ACD mice (n = 38 neurons for sham, n = 40 neurons for ACD mice). **(C)** Resting membrane potential showed no difference (n = 30 neurons for sham, n = 49 neurons for ACD mice). **(D)** Typical traces of action potential from Calcrl^+^ neurons in Sham or ACD mice. (E-I) Action potential waveform analysis demonstrated (n = 32 neurons for each group): Reduced threshold potential **(E)**; Elevated AP peak amplitude **(F)**. Unchanged maximal rise slope **(G)**; Reduced half-width **(H)**; Decreased AHP amplitude **(I)**; Increased number of spikes upon 100 pA depolarization **(J)**. Data are represented as mean ± SEM (n = 5 mice per group). ns: not significant, *p < 0.05, **p < 0.01. Differences between the groups were statistically evaluated using a two-tailed unpaired Student’s t-test.

To elucidate the cellular mechanisms underlying chronic itch pathogenesis, we compared electrophysiological properties of spinal Calcrl^+^ neurons between chronic itch model mice and controls. Rheobase measurements revealed significant hyperexcitability of spinal Calcrl^+^ neurons in ACD model (Sham: 41.83 ± 3.81 pA vs. ACD: 28.75 ± 3.54 pA; p = 0.0464, n = 38 neurons from 5 mice for sham, group, n = 40 neurons from 5 mice for ACD group; Fig 3A and 3B), despite unaltered resting membrane potential (Sham: −57.14 ± 1.13 mV vs. ACD: 55.64 ± 1.07 pA; p = 0.3579, n = 30 neurons from 5 mice for sham group, n = 49 neurons from 5 mice for ACD group; Fig 3C). Action potential waveform analysis of Calcrl^+^ neurons demonstrated four key alterations in ACD group (Figs 3D-J, n = 32 neurons from 5 mice/group): (1) Reduced threshold potential (−21.98 ± 1.42 mV vs. −25.95 ± 1.16 mV; p = 0.0342); (2) Elevated AP peak amplitude (58.19 ± 2.28 mV vs. 65.30 ± 2.57 mV; p = 0.0426), (3) Unchanged maximum rise slope (62.06 ± 6.74 mV/ms vs. 73.89 ± 8.58 mV/ms, p = 0.2822); (4) Reduced half-width (1.23 ± 0.06 ms vs. 0.97 ± 0.06 ms; p* =* 0.0022); (5) Decreased after hyperpolarization (AHP) amplitude (33.01 ± 2.29 mV vs. 27.62 ± 1.38 mV; p = 0.3340). Furthermore, the firing rate of APs evoked by 100 pA, 1-s depolarizing currents was significantly elevated in ACD mice compared to sham controls (19.27 ± 2.75 vs. 30.26 ± 3.75 spikes/s; p = 0.0221; Fig 3J). These electrophysiological signatures collectively demonstrate that chronic itch enhances intrinsic excitability of spinal Calcrl⁺ neurons through AP waveform remodeling and threshold AP reduction.

### Plasticity of mechanical itch signaling pathways in chronic itch models

**Fig 4:**
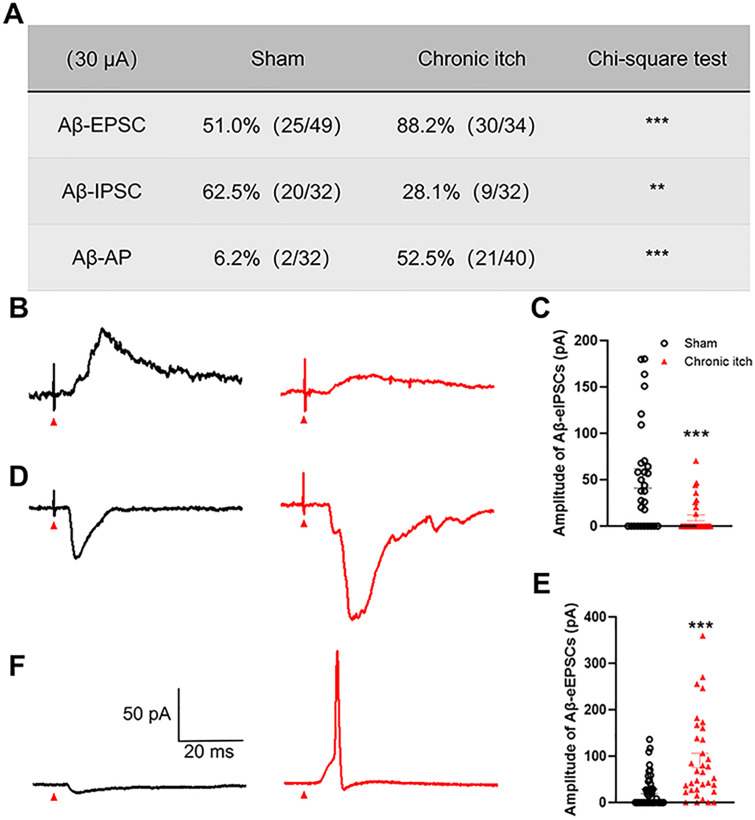
Synaptic plasticity in spinal Calcrl^+^ neurons under chronic itch condition. **(A)** Summarized table of Aβ-evoked IPSCs, EPSCs, and APs in Sham and chronic itch mice. Statistical analysis: chi-square test. **(B)** Representative traces showing Aβ-evoked IPSCs of spinal Calcrl^+^ neurons from Sham (black trace) and ACD (red trace) mice. **(C)** Quantification revealed significantly reduced IPSC responses in chronic itch mice. Group differences were statistically evaluated using a two-tailed unpaired Student’s t-test. **(D)** Representative traces for Aβ-induced EPSCs of spinal Calcrl^+^ neurons from Sham (black) and ACD (red) mice. **(E)** Quantification revealed EPSC responses were significantly increased in chronic itch mice. Differences between the groups were analyzed by a two-tailed unpaired Student’s t-test. **(F)** Typical traces for Aβ-induced IPSP or AP of spinal Calcrl^+^ neurons from Sham (black) and ACD (red) mice. Data are presented as mean ± SEM (n = 5 mice per group). **p < 0.01, ***p < 0.001. Red arrowheads denote stimulation artifacts.

Building on our prior work [17], we investigated synaptic plasticity in spinal Calcrl+neurons. Aβ-fiber-evoked inhibitory postsynaptic currents (Aβ-eIPSCs) exhibited marked suppression: Aβ-eIPSCs were detected in 62.5% of Calcrl^+^ neurons from Sham group (20/32) versus 28.13% in ACD mice (9/32; chi-square test: χ^2^(1) = 7.630, p = 0.0057; Fig 4A). Correspondingly, IPSC amplitude decreased significantly from 46.34 ± 9.47 pA (Sham) to 8.88 ± 3.15 pA (ACD; two-tailed unpaired Student’s t-test: p = 0.0004; Figs 4B and 4C). In contrast, Aβ-fiber-evoked excitatory postsynaptic currents (Aβ-eEPSCs) showed pronounced potentiation (Fig 4A): Aβ-eEPSCs were observed in 51.02% of Calcrl^+^ neurons from Sham group (25/49) versus 88.23% in ACD mice (30/34; chi-square test: χ^2^(1) = 12.44, p = 0.0004). EPSC amplitude increased >3.7 fold from 23.93 ± 6.72 pA (Sham) to 90.65 ± 16.43 pA (ACD; two-tailed unpaired Student’s t-test: p < 0.0001, Figs 4D and 4E).

Electrophysiological characterization further revealed hyperexcitable network properties: Aβ-fiber-evoked AP firing probability in Calcrl^+^ neurons surged from 6.25% (Sham: 2/32) to 52.5% (ACD: 21/40; chi-square test: χ^2^(1) = 17.49, p < 0.0001; Figs 4A and 4F). These data collectively demonstrate maladaptive remodeling of Aβ-fiber synaptic circuitry in chronic itch models, characterized by concurrent inhibitory deficit and excitatory potentiation.

## Discussion

The spinal dorsal horn (SDH) serves as a central node for sensory information processing, where the heterogeneous functional properties of excitatory and inhibitory interneurons are fundamental for accurately differentiating distinct sensory modalities, including pain and itch [6,25].

Chemical itch initiates with peripheral itch-sensing C-fibers expressing Mas-related G protein-coupled receptors (Mrgprs; e.g., MrgprA3, MrgprD), natriuretic peptide B (Nppb) or neuromedin B (NMB), which detect pruritogenic stimuli at the skin level and relay these signals to superficial SDH interneurons specialized for chemical itch processing [26–29]. Within the SDH, neurons expressing neuromedin B receptor (NMBr) or Nppb receptor (Npra) are activated by these peripheral peptides. Notably, Npra^+^ SDH interneurons co-express gastrin-releasing peptide (GRP)—another critical pruritogenic neuropeptide [28–31]. Peripheral release of Nppb specifically activates Npra on these neurons, triggering GRP release. This spinal GRP then acts on its receptor, gastrin-releasing peptide receptor (GRPR), subsequently transmit itch signals to spinal NK1R (also known as Tacr1)-expressing projection neurons, which ultimately convey the information to supraspinal centers [24,28,30,32,33]. Peripheral and spinal somatostatin (SOM)^+^ neurons have also been implicated in the modulation of chemical itch [34,35], while a subset of Bhlhb5^+^ inhibitory interneurons (B5-I neurons, releasing the kappa opioid dynorphin)—specifically those co-expressing galanin (GAL) or enkephalin (ENK)—suppress GRPR^+^ neurons to dampen itch transmission [35,36].

Mechanical itch processing, in parallel, relies on distinct circuit circuits. Spinal Ucn3^+^/NPY1R^+^ neurons, identified as key mechanical itch transmitters, receive monosynaptic input from peripheral Toll-like receptor 5^+^ low-threshold Aβ-mechanoreceptors (TLR5^+^ Aβ-LTMRs) and are gated by NPY^+^ inhibitory interneurons [17,18]. Beyond this foundational circuit, the NPY system exhibits broad functional versatility: studies reveal it not only modulates both mechanical and histaminergic itch [37] but also influences spinal neuropathic pain processing [38]. Delving deeper into its downstream mechanisms, NPY signaling exerts analgesic effects in neuropathic pain via NPY1R-expressing interneurons that coexpress gastrin-releasing peptide (GRP) [14,15]. Intriguingly, recent insights further reveal a critical intersection of this system with pruritus: opioid drugs induce itch by suppressing the gating of GRP^+^ neurons by NPY^+^ neurons, thereby disinhibiting the GRP-GRPR itch circuit—a disruption that directly triggers pruritic responses [39].

**Fig 5:**
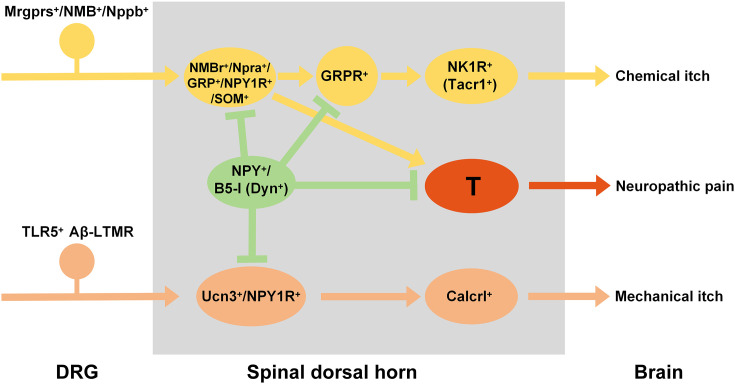
Schematic showing neural circuits in spinal dorsal horn mediating sensory modalities.

Concurrently, spinal Calcrl^+^ neurons have emerged as critical players in mechanical itch transmission, directly projecting to FoxP2^+^ neurons in the parabrachial nucleus (PBN) to initiate itch-related behaviors [24]. Beyond their role in itch, emerging evidence further demonstrates that spinal Calcrl^+^ neurons also mediate the transmission of cool sensation signals, expanding their functional repertoire in sensory processing [40]. Collectively, these observations underscore the functional complexity of SDH: specialized interneuron populations coordinate to segregate and transduce diverse sensory modalities, enabling precise coding of itch versus pain and other tactile inputs (Fig 5).

A hypothetic model depicting specialized interneuron networks in the spinal dorsal horn that differentially process chemical itch, mechanical itch, and neuropathic pain. Chemically evoked itch signals originate from DRG neurons expressing Mrgprs, Nppb NMB. Upon detecting pruritogenic stimuli, these DRG neurons release NMB or Nppb, which activate their respective spinal receptors—NMBr or Npra—on interneurons. Spinal interneurons co-expressing NMBr/Npra and GRP relay the signal: GRP released from these neurons activates GRPR^+^ interneurons, which then transmit chemical itch information to NK1R^+^ projection neurons. This circuit can be facilitated by SOM⁺ neurons and gated by B5-I neurons (Dyn^+^). Mechanical itch is mediated by peripheral TLR5 ⁺ Aβ-LTMRs, whcich activate spinal Ucn3 ⁺ /NPY1R⁺ interneurons (gated by NPY⁺ interneurons). These Ucn3 ⁺ /NPY1R⁺ interneurons then relay signals to downstream Calcrl⁺ neurons, which project to supraspinal targets. Spinal NPY1R^+^ neurons, which co-express GRP, further regulate both GRP-GRPR itch circuits and neuropathic pain via NPY signaling. Together, heterogeneous excitatory/inhibitory interneurons in SDH thus coordinate to segregate sensory inputs (itch vs. pain) with precision. T: transmission neuron.

While our understanding of the spinal itch circuits has advanced, chronic itch remains a therapeutically challenging condition due to its multifactorial pathophysiology. A central unresolved question is: How do these neurons dynamically encode the hypersensitivity characteristic of chronic itch? Our present discovery—that spinal Calcrl⁺ neurons act as critical amplifiers of mechanical itch signals in chronic itch—fundamentally reshapes our understanding of itch neurocircuitry.

Our study now reveals a functional metamorphosis in Calcrl^+^ neurons during chronic itch, whereby these neurons transition into pathological amplifiers of itch signals. This transformation is defined by three distinct features that distinguish them from their acute counterparts: asymmetric synaptic remodeling, hyperexcitable intrinsic properties, and maladaptive network integration. These findings bridge a critical gap between the anatomical framework and the pathophysiological mechanisms driving chronic itch.

Specifically, under chronic itch condition, spinal Calcrl^+^ neurons undergo a fundamental functional remodeling characterized by a near four-fold increase in Aβ-evoked EPSC amplitude and 76% reduction in IPSC efficacy, marking a shift from balanced synaptic transmission to excitation-dominant signaling. Notably, this functional remodeling is accompanied by elevated intrinsic excitability of spinal Calcrl⁺ neurons—a key distinction from acute itch models, where Calcrl^+^ neurons primarily transmit sensory inputs without synaptic amplification. This disruption of the excitatory-inhibitory (E-I) balance likely arises from concerted presynaptic and postsynaptic adaptations that collectively potentiate excitatory synaptic transmission under chronic itch conditions.

Presynaptic hyperactivity in chronic itch may arise from the following mechanisms: **(1) Elevated glutamate release probability:** Increased action potential (AP) frequency in upstream circuits (as shown in our prior study of mechanical itch transmission neurons under chronic itch [17]) potentiates glutamate release. Lee et al. further revealed that Ucn3 ⁺ neurons exhibit enhanced excitability via ion channel remodeling (e.g., Nav1.6/Cav2.3 upregulation) [41], which may further elevate presynaptic glutamate release. **(2) Enhanced glutamate vesicle priming:** Hyperactivity presynaptic terminals upregulate key proteins involved in glutamate vesicle docking/priming (e.g., Munc13, syntaxin, SNAP-25) at presynaptic terminals, expanding the readily releasable pool (RRP) of vesicles and increasing EPSC frequency/amplitude [42,43]. **(3) Impaired presynaptic inhibition:** Chronic itch weakens inhibitory gating on mechanical itch-transmitting neurons [17]. Notably, Lee et al. demonstrated that NPY⁺ neurons exhibit reduced excitability due to downregulation of ion channels such as Nav1.6 [41]. Additionally, impaired GABA synthesis and release—such as that caused by altered glutamate decarboxylase 65/67 (GAD65/67) expression, specifically reduced membrane translocation of GAD65/67 enzymes—may further compromise presynaptic inhibition [44]. Our electrophysiological recordings confirm diminished inhibitory input to Calcrl⁺ neurons in chronic itch, which likely involves GABAergic plasticity.

Beyond presynaptic adaptations, postsynaptic mechanisms may also contribute to this E-I imbalance in chronic itch through the following key processes: **(1) Chloride homeostasis disruption:** Dysfunction of GABA_A_ receptors—ligand-gated Cl⁻ channels, reduces intracellular Cl⁻ influx [45–47]. Concurrently, K⁺-Cl⁻ cotransporter-2 (KCC2) dysfunction—critical for maintaining transmembrane chloride gradients—leads to a shift in the anion gradient, converting normally inhibitory anionic synaptic currents to excitatory ones [48–52]. **(2) AMPA receptor (AMPAR) trafficking/upregulation:** Pathological plasticity alters AMPAR surface expression (e.g., GluA1 subunits) or phosphorylation state, promoting membrane insertion and enhancing conductance/glutamate-binding to amplify EPSCs [53–56]. **(3) N-methyl-d-aspartate (NMDA) Receptor (NMDAR) activation:** Pathological states enhance NMDAR function via Mg² ⁺ block reduction (altered membrane potential/intracellular Mg² ⁺ levels) or increased trafficking, prolonging EPSC decay kinetics and increasing charge transfer to facilitate excitatory signaling [57–60].

Collectively, the presynaptic and postsynaptic adaptations underlying the transition of Calcrl⁺ neurons from balanced relays to pathological amplifiers—driven by excitatory dominance and suppressed inhibition—provide a mechanistic framework for mechanical itch hypersensitivity in chronic itch.

In this study, we demonstrate that spinal Calcrl⁺ neurons contribute to itch sensitization across multiple chronic itch models (ACD, AD, PSO), but distinct immune mechanisms underlying these models raise a critical question: Do the observed synaptic/excitability changes arise from convergent neural plasticity or model-specific upstream signals?

ACD, AD, and PSO are distinguished by distinct immune signatures: ACD is characterized by Th1/Th17-mediated type IV (delayed) hypersensitivity [61–63]; AD features Th2-skewed immune responses and impaired skin barrier function [64,65]; and PSO reflects Th17/IL-23 axis-driven autoimmunity [66–68]. Despite their distinct model-specific immune drivers, these three conditions may converge on shared mechanisms that sensitize Calcrl⁺ neurons: Chronic neuroinflammation induced by these immune processes across models could engage conserved pathways, where pro-inflammatory mediators released during inflammation could disrupt inhibitory control or enhance excitatory signaling via the presynaptic and postsynaptic mechanisms described above, collectively amplifying excitatory postsynaptic currents (EPSCs). Nevertheless, whether these proposed mechanisms are truly conserved across models or instead represent model-specific adaptations remains to be confirmed.

Given the current reliance on inference, future studies must directly compare how immune-mediated pathways regulate Calcrl⁺ neuron excitability—via gene expression, synaptic protein dynamics (e.g., AMPAR, NMDAR, AMPAR, KCC2), and electrophysiology—to resolve shared versus unique contributions to chronic itch.

Therapeutically, our validation of Calcrl^+^ neurons as a druggable target via chemogenetic inhibition holds promise. However, questions remain: Do Calcrl^+^ neurons interact with histaminergic or non-histaminergic itch? Is IPSC reduction stems from structural loss of inhibitory synapses (via spine pruning) or functional suppression (e.g., KCC2 downregulation impairing Cl^-^ homeostasis). Resolving these could illuminate novel therapeutic targets—such as calcrl-dependent transcription factors that coordinately regulate excitation-inhibition balance—to restore functional circuit architecture in chronic itch.

In summary, this study resolves a critical gap in itch neurocircuitry by defining a spinal Calcrl-dependent excitatory pathway that drives mechanical itch sensitization. Targeting this pathway could offer precision therapies for patients refractory to current treatments.

## Conclusions

Our findings demonstrated the spinal Calcrl^+^ neuron as a central amplifier of mechanical itch sensitization. By linking Calcrl^+^ neuron hyperexcitability, enhanced excitatory synaptic transmission, and diminished inhibitory control to the development and maintenance of chronic itch, our study fills a critical gap in the field. These findings not only advance our mechanistic understanding of chronic itch but also provide actionable targets for disrupting maladaptive itch circuits, paving the way for the development of novel, targeted therapies for this underserved dermatological disorder.
